# Mechanisms of fitness cost reduction for rifampicin-resistant strains with deletion or duplication mutations in ***rpoB***

**DOI:** 10.1038/s41598-018-36005-y

**Published:** 2018-11-30

**Authors:** Gerrit Brandis, Diarmaid Hughes

**Affiliations:** Uppsala University, Department of Medical Biochemistry and Microbiology, Uppsala, 75123 Sweden

## Abstract

Rifampicin resistance (Rif^R^) is caused by mutations in *rpoB*, encoding the β-subunit of RNA polymerase. Rif^R^ mutations generally incur a fitness cost and in resistant isolates are frequently accompanied by compensatory mutations in *rpoA*, *rpoB* or *rpoC*. Previous studies of fitness compensation focused on Rif^R^ caused by amino acid substitutions within *rpoB*. Rif^R^ is also caused by deletion and duplication mutations in *rpoB* but it is not known whether or how such mutants can ameliorate their fitness costs. Using experimental evolution of *Salmonella* carrying Rif^R^ deletion or duplication mutations we identified compensatory amino acid substitution mutations within *rpoA*, *rpoB* or *rpoC* in 16 of 21 evolved lineages. Additionally, we found one lineage where a large deletion was compensated by duplication of adjacent amino acids (possibly to fill the gap within the protein structure), two lineages where mutations occurred outside of *rpoABC*, and two lineages where a duplication mutant reverted to the wild-type sequence. All but the two revertant mutants maintained the Rif^R^ phenotype. These data suggest that amino acid substitution mutations are the major compensatory mechanism regardless of the nature of the primary Rif^R^ mutation.

## Introduction

Rifampicin is a bactericidal antibiotic that is part of the short-course anti-tuberculosis treatment^1^. Resistance to rifampicin can be caused by any of over 100 distinct changes in the β-subunit of the bacterial RNA polymerase (RNAP)^2^ where rifampicin binds and exerts its function by inhibiting transcription^3^. The majority of these mutations cause a significant fitness cost to the bacteria^2^ and recent studies using *Salmonella* as a model organism and/or genome sequences of clinical MTB isolates have shown the importance of fitness-compensatory evolution in the development of clinical rifampicin resistance^2,4–7^. These studies have shown that the initial fitness cost caused by a Rif^R^ mutation can be ameliorated by second-site mutations within the RNAP genes *rpoA*, *rpoB* or *rpoC* that encode the α-, β- and β’-subunit of the RNAP.

The majority of rifampicin-resistant clinical MTB isolates are found to have amino acid substitutions in the rifampicin resistance determining region (RRDR)^8–10^ but a number of deletions and duplication within this region can give rise to rifampicin resistance^2^ and these deletions and duplications are also found in clinical isolates^11^. So far, all studies on compensatory evolution have focused on the amino acid substitution mutations and there is no data on the evolution of strains carrying Rif^R^ deletions and duplications. It is not clear if these mutational types would follow the same evolutionary trajectories as the amino acid substitutions. Deletions or insertions of multiple amino acids are expected to cause more drastic changes to the protein structure compared to substitutions and the fitness cost associated to these changes might therefore not be ameliorated as easily.

In this study, we tested this hypothesis by evolving six Rif^R^ deletion mutants and one Rif^R^ duplication mutant with selection for increased fitness. Our aims were: (i) to identify growth-compensatory mutations, and (ii) to compare the compensatory evolution of Rif^R^ deletions and duplications to the compensatory evolution of Rif^R^ amino acid substitutions.

## Results and Discussion

### Characterization of RifR mutations and compensatory evolution

**Figure 1:**
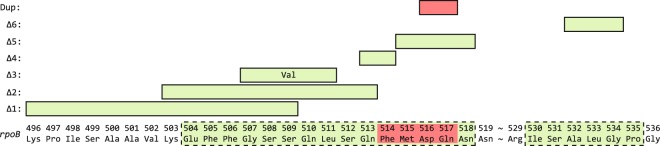
Overview of mutations analysed in the study. Amino acids and positions of the relevant *rpoB* gene segment are shown. Amino acids that can result in rifampicin resistance when deleted are boxed in green with a dashed line, and amino acids where duplications have been linked to rifampicin resistance are shown in red. The Rif^R^ deletion and duplication mutations used in this study are indicated above the sequence. Green boxes indicate amino acids that are deleted and the red box indicates duplicated amino acids (D516, Q517). The ∆1 deletion allele carries additionally a *rpoB* Q490P mutation. The ∆3 deletion fuses parts of codon 507 and 511 creating a valine codon.

There are two regions within the RNA polymerase β-subunit RRDR where deletions can give rise to rifampicin resistance (amino acids 504–518 and 530–535)^2^. We selected five different Rif^R^ deletions that cover all amino acids within the first, larger region and one deletion that cover three out of the five amino acids in the second region (Fig. 1). Rif^R^ duplication mutations are mainly located within a stretch of four amino acids (amino acids 514–517)^2^ and we selected a duplication of two of these amino acids (Fig. 1). All but one of these Rif^R^ alleles cause a high rifampicin MIC of 3000 mg/L (wild-type: 12 mg/L, *rpoB* ∆532–534: 500 mg/L) and the growth fitness of the Rif^R^ isolates varies from 0.23 to 0.53 relative to the sensitive wild type (Table 1).

**Table 1:** Compensatory mutations found in the RNAP genes.

Mutations in *rpoA*, *rpoB* or *rpoC*		Rifampicin MIC (mg/L)	Relative fitness ± SD^a^
Rif^R^ (*rpoB*)	Compensatory		
wild-type	isogenic wild-type	12	1.00 ± 0.02
∆496–509 + Q490P	unevolved parental strain	3000	0.27 ± 0.01
∆496–509 + Q490P	*rpoB* S512F	3000	0.72 ± 0.01
∆496–509 + Q490P	*rpoB* D483ALGD	3000	0.55 ± 0.01
∆496–509 + Q490P	*rpoB* S512F	3000	0.72 ± 0.01
∆504–513^b^	unevolved parental strain	3000	0.23 ± 0.00
∆504–513^b^	*pdxB* L347L (CTG > TTG), *hin* inversion	3000	0.56 ± 0.01^c^
∆504–513^b^	*rpoB* S499F	3000	0.52 ± 0.01
∆504–513^b^	*rpoB* E142A, *barA* Q470*	3000	0.51 ± 0.00
∆507–511- > V	unevolved parental strain	3000	0.53 ± 0.00
∆507–511- > V	*rpoC* L175Q	3000	0.69 ± 0.02
∆507–511- > V	*rpoB* S512F	3000	0.85 ± 0.02
∆507–511- > V	*rpoA* R195P	3000	0.84 ± 0.02
∆513–514	unevolved parental strain	3000	0.28 ± 0.07
∆513–514	*rpoB* G713C	3000	0.68 ± 0.03
∆513–514	*rpoB* D516G	3000	0.60 ± 0.01
∆513–514	*rpoB* S512P	3000	0.69 ± 0.01
∆515–518	unevolved parental strain	3000	0.37 ± 0.00
∆515–518	*rpoC* L770P	3000	0.64 ± 0.03
∆515–518	*rpoB* E523D	3000	0.76 ± 0.02
∆515–518	*rpoB* H526L	3000	0.78 ± 0.01
∆532–534^b^	unevolved parental strain	500	0.46 ± 0.00
∆532–534^b^	*rpoC* T674I	500	0.75 ± 0.01
∆532–534^b^	*rpoC* D806A, *STM14_1615* G-56A	500	0.76 ± 0.01
∆532–534^b^	*dksA* G-86A, *STM14_1020* D165G	500	0.68 ± 0.01^c^
DQ(516–517)DQDQ	unevolved parental strain	3000	0.41 ± 0.01
DQ(516–517)DQDQ	*rpoB* DQ(516–517)DQNQ	3000	0.71 ± 0.02
DQ(516–517)DQDQ	reversion	12	0.98 ± 0.03^c^
DQ(516–517)DQDQ	reversion	12	1.01 ± 0.02^c^

^a^Fitness ± standard deviation relative to the isogenic wild type.

^b^Isolates with whole genome sequence data.

^c^Compensatory mutations outside *rpoA*, *rpoB* and *rpoC* and reversion mutations were not reconstructed. Relative fitness of evolved isolate is shown.

Three independent lineages of each of the seven Rif^R^ isolates were evolved by serial passage in LB medium with selection for increased growth rates and after 100 generations, diluted cultures were plated on LA agar (approximately 100 colonies per plate). In each evolved lineage, all observed colonies were significantly larger than colonies of the unevolved parental strain indicting that isolates with fitness compensatory mutations had swept the populations. The colonies within each plate were approximately uniform in size so that only a single clone of each lineage was selected for further analysis.

### Identification and characterization of compensatory mutations

We sequenced the *rpoA*, *rpoB* and *rpoC* genes of all 21 evolved isolates to identify putative compensatory mutations. Seventeen of the evolved isolates had acquired a secondary mutation within one of the sequenced RNAP genes (1 in *rpoA*, 12 in *rpoB* and 4 in *rpoC*), two isolates had acquired reversion mutations that segregated the duplication back to the single-copy wild-type sequence, and two isolates retained the original Rif^R^ mutation within *rpoB* but had not acquired a secondary mutation in any of the three sequenced genes. Interestingly, half of the secondary mutations acquired in *rpoB* (6 out of 12) are known to be rifampicin resistance mutations by themselves^2^. No rifampicin was present in the growth medium during the evolution experiments and there is no expectation of any selection for additional resistance mutations. However, this phenomenon of selecting compensatory mutations that are themselves resistance mutations has been observed in several independent studies of compensatory evolution of Rif^R^ substitution mutations^4–7^. This observation implies a strong epistatic fitness landscape involving many different pairs of RifR mutations, a phenomenon that has been studied extensively in *Pseudomonas*^12^.

We reconstructed the 17 secondary RNAP mutations in the ancestral backgrounds and measured relative fitness and rifampicin MIC. The results show that each of the secondary mutations is necessary and sufficient to increase the growth rate of the ancestral Rif^R^ strains (Table 1). For the two strains with no secondary mutation within the RNAP genes and the two strains with reversion mutations, we determined relative fitness and rifampicin MIC in the evolved strain. Unsurprisingly, the two isolates with reversion mutations were indistinguishable from the wild type in both relative fitness and rifampicin MIC (Table 1). The two isolates without secondary mutations within the RNAP genes displayed increased fitness indicating the presence of either compensatory mutations outside the sequenced RNAP genes or mutations to adapt to growth under laboratory conditions^13^. With the exception of the revertants none of the remaining 19 evolved strains showed any change in rifampicin MIC (Table 1).

We decided to whole genome sequence the two isolates with compensatory mutations outside the RNA polymerase genes to identify what kind of mutations might be responsible for the increase in growth rate. The results (Table 1) show that each of these two isolates had acquired two genetic changes. One isolate (*rpoB* ∆504–513) had inverted the *hin* region relative to the parental strain leading to a change in flagellin expression^14^, and also acquired a synonymous mutation within the *pdxB* gene. Interestingly, the *pdxB* genes overlaps with the flagella biosynthesis regulator *flk* suggesting the possibility that the synonymous mutation might affect the expression of *flk*. Changes in flagella synthesis have previously been linked to the adaptation of *Salmonella* to growth in LB medium^13^. It is therefore possible that the two mutations found in this isolate represent adaptation to growth in this laboratory medium rather than being specific adaptations to the cost caused by the rifampicin resistance mutation. The second isolate (*rpoB* ∆532–534) acquired a mutation in the putative transcriptional regulator STM14_1020 and a mutation upstream of the transcriptional regulator *dksA*, potentially affecting *dksA* expression. Both mutated genes are transcriptional regulators with diverse potential downstream effects. It is therefore not possible to distinguish whether these mutations are selected as an adaptation to the rifampicin resistance mutation, and/or to growth under laboratory conditions. To test the prevalence of mutational adaptation to growth under laboratory conditions on the evolved Rif^R^ isolates that had acquired compensatory mutations in the RNAP genes, we also sequenced the genomes of the parallel evolved lineages from the same respective ancestral isolates. Two of the four evolved isolates had no mutation outside those identified within the RNAP genes. The third isolate had acquired a mutation upstream STM14_1615, a gene annotated only as encoding a hypothetical protein and not previously associated with any phenotype (Table 1). The fourth isolate had acquired a mutation outside the RNAP genes (*barA* Q470*) that has previously been shown to be adaptive to growth in LB medium^13^. These results indicate that adaptation to growth under laboratory condition is only a minor contributing factor in those lineages where compensatory mutations to the fitness costs of Rif^R^ mutations were selected within the RNA polymerase genes, at least during the timespan of the evolutionary experiment (100 generations).

### Locations of compensatory mutations in RNA polymerase

Our results show that the majority of evolved isolates acquire compensatory mutations within the RNAP genes. Some of these mutations have previously been shown to compensate for the fitness cost caused by Rif^R^ amino acid substitutions in *rpoB*. The *rpoB* D516G and *rpoC* L770P mutations were shown to compensate the effects of the Rif^R^ mutation *rpoB* R529C^4^ and the mutation *rpoC* T674I can compensate the effects of the Rif^R^ mutations S531L^5^. Other mutations are located in regions of the RNAP genes that have been linked to compensatory mutations. The *rpoA* R195P mutation for example is adjacent to the compensatory mutations *rpoA* Q194P and *rpoA* T195S^4,5^. All but one (*rpoB* S512F) of the compensatory mutations within the RNAP genes were isolated as singletons within our evolution experiment. This is consistent with the existence of a large pool of potential compensatory mutations. The locations of the compensatory mutations are in full agreement with previous studies that show that mutations that compensate the fitness cost of Rif^R^ amino acid substitutions are clustered in particular structural regions of the RNA polymerase, e.g. the RpoA-RpoC interface^4,7^. These observations are consistent with the hypothesis that structural changes leading to fitness compensation in this region of RNAP can be achieved by many different combinations of mutations.

**Figure 2:**
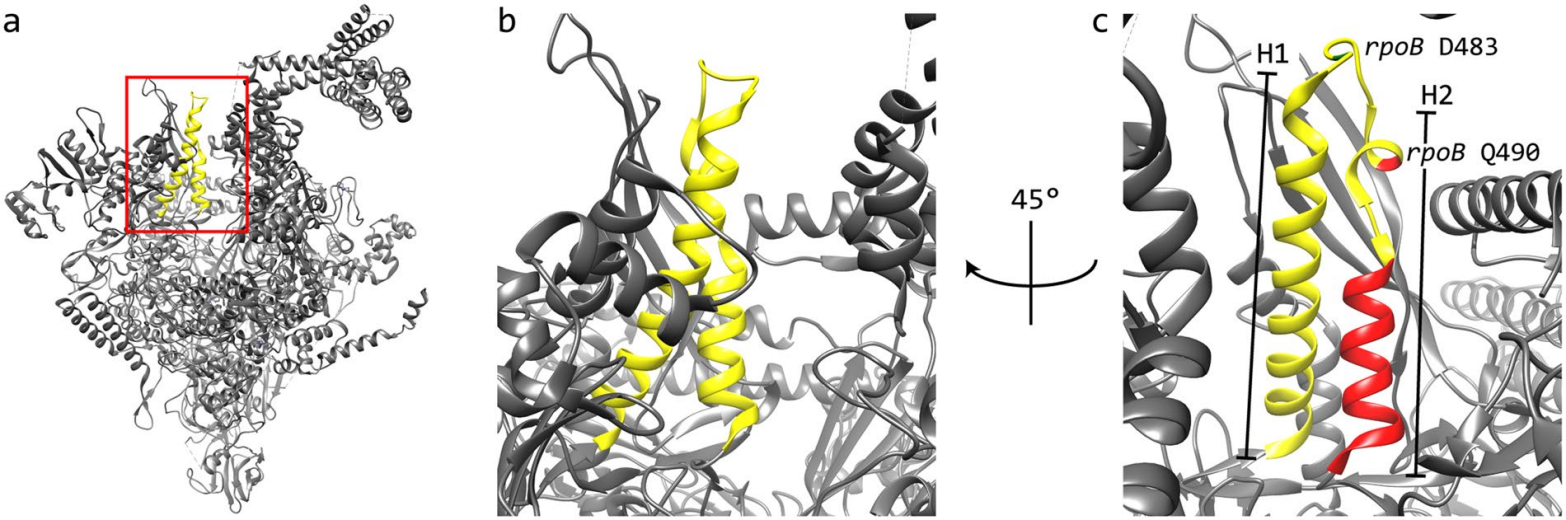
Structural analysis of the “gap repair” mechanism. (**a**) The RNA polymerase holoenzyme of *Escherichia coli*, PDB code 4MEY. The affected helix-turn-helix domain is shown in yellow. (**b**) Close up of the affected helix-turn-helix domain region outlined by the red box in panel a. (**c**) Rotated and annotated view. Amino acids that were affected by the initial Rif^R^ mutation (*rpoB* ∆496–509, Q490P) are shown in red and the location of the compensatory insertion (*rpoB* D483ALGD) is shown in green.

Interestingly, one of the isolates with the 14 amino acid long deletion acquired a three amino acid insertion close to the deletion (Table 1). Insertions like this have not been seen in the evolution of amino acid substitution and might represent a type of “gap repair” mechanism in which the deleted part of the protein is replaced with a new, and possibly relatively random, amino acid sequence. The large deletion (*rpoB* ∆496–509) is located within a helix-turn-helix domain of the RNA polymerase (Fig. 2). The affected domain consists of two helices (H1: *rpoB* 455–481, H2: *rpoB* 488–509) and the Rif^R^ deletion *rpoB* ∆496–509 leads to the deletion of a large part of the H2 helix (Fig. 2c). This deletion will most likely result in a distortion of the H1 helix and other closely located regions of the RNA polymerase due to the shortened H2 helix and these distortions could explain the large fitness cost of the deletion (73% relative to the wild-type). Interestingly, the large deletion was isolated together with a *rpoB* Q490P mutation. This mutation leads to a double proline motive which will most likely break the remaining part of the H2 helix and thereby lead to a partial relief of the RNAP distortions. The initial deletion leads to a gap of about 21.5 Å in the protein structure and breaking the remainder of the H2 helix would extend its length by around 11.5 Å leaving a 10 Å gap. The addition of three amino acids in the compensated mutant would extend the coil by approximately 10.5 Å and thereby fill the gap created in the protein structure. This could explain how the addition of three amino acids compensates for the deletion of fourteen amino acids. The resulting helix-turn-coil structure would fill the same space within the protein structure but lack the H2 helix, which is likely responsible for the remaining fitness cost in the compensated mutant (45% relative to the wild-type).

### Deletion/duplications mutations in *rpoB* in Rif^R^ MTB isolates

We conducted a literature search for published sequencing results of clinical MTB isolates that show the presence of deletions or duplications in combination with secondary mutations in *rpoB*, as previously done for Rif^R^ substitution mutations^2,6,7^. This search was complicated by two factors: (i) deletions and duplications are rare in clinical isolates, and (ii) many publications that describe deletions or duplications have only sequenced the RRDR of the *rpoB* gene so that only secondary mutations within this region could be identified. Nevertheless, we identified four clinical MTB isolates with a combination of deletion and substitution mutations in *rpoB* (*rpoB* ∆509 + S531L^15^, *rpoB* ∆509 + Q513L + S531L^15^, *rpoB* ∆527 + L524Y + T525P + H526Q^11^ and *rpoB* ∆522–525 + H526D + S531L^11^) and two isolates with duplications in combination with a substitution in *rpoB* (*rpoB* F514FF + S531L^16^ and *rpoB* F514FR + L533P^17^). While the occurrence of these double mutations in *rpoB* is consistent with a compensatory mechanism it does not constitute a proof of selection for compensation (additional experimental evidence would be required) and it does not distinguish whether any compensation was selected by growth fitness costs, or by other forces such as selection to escape immune pressure or other host adaptation processes. However, the number of whole genome sequences of clinical MTB isolates is growing rapidly and this data will increase the potential to quantitatively assess the significance of secondary mutations in RNAP and their potential role in determining the success of clinical isolates.

## Conclusions

**Figure 3:**
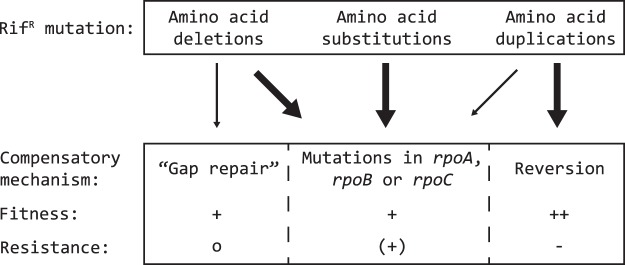
Overview of evolutionary trajectories. Fitness-compensatory mechanisms for the different types of rifampicin resistance mutations. An increase in bacterial fitness is annotated with a ‘+’ and an increase back to wild-type fitness with a ‘++’. Changes in rifampicin resistance levels are annotated as ‘−’ (reduced back to wild-type levels), ‘o’ (no change) and ‘(+)’ (has the potential to increase resistance).

Compensatory mutations that improve the growth rate of mutants with deletions or a duplication within the β-subunit of RNAP are found as amino acid substitution mutations in each of the three RNAP genes, *rpoA*, *rpoB* and *rpoC*. An exception to this rule was a lineage that carried a deletion of 14 amino acids and that compensated the loss of sequence by the duplication of adjacent amino acids, most likely to fill the gap created by the Rif^R^ deletion (Fig. 2). In addition, the Rif^R^ duplication mutant could be compensated by amino acid substitution mutations within the *rpoB* gene but it also frequently reverted back to the rifampicin sensitive wild-type sequence (Fig. 3). In conclusion, the evolutionary trajectories for rifampicin-resistant *rpoB* alleles with deletions are almost identical to those of rifampicin-resistant *rpoB* alleles with amino acid substitutions.

## Methods

### Bacterial strains and growth conditions

All strains are derived from the virulent *Salmonella enterica* serovar Typhimurium strain 14028s^18^ and have previously been described^2^ or were constructed by oligonucleotide recombineering^19^. Bacteria were grown with shaking at 37 °C in Luria broth (LB) or on LA plates (LB solidified with 1.5% agar, Oxoid). Genetic markers were moved between strains using P22 HT phage^20^. Growth rates were measured using a Bioscreen C machine (Oy Growth curves Ab Ltd). Three independent cultures per strain were grown overnight in LB after which they were diluted 3000-fold in fresh LB. 300 µL of each dilution were incubated at 37 °C with continuous shaking in honeycomb microtiter plates, with optical density readings at 5 min intervals. Doubling times were calculated from the increase in optical density at 600 nm.

### Minimum inhibitory concentration measurements

Rifampicin MIC values up to a concentration of 32 mg/L were determined using E-test (BioMérieux, France) on LA plates. Rifampicin MIC values above 32 mg/L were measured by broth dilution in microtiter plates. Three independent cultures per strain were grown overnight in LB after which they were diluted 1000-fold in fresh LB. 10 µL volumes of dilutes cells were inoculated into microtiter plate wells with 190 µL LB (final cell concentration of 10^5^ cfu/mL) containing rifampicin to give final concentrations of 50, 100, 150, 250, 500, 750, 1500 and 3000 mg/L. Cultures were incubated for 18 h at 37 °C.

### Evolution by serial passage

Three independent lineages of each parental strain were grown overnight in 15 mL tubes with shaking at 37 °C in 2 mL LB. Each lineage was serially passaged after each growth cycle by transferring 2 µL of culture into 2 mL fresh LB to initiate the next cycle. All evolutions were carried out for 10 cycles (100 generations) after which a single clone per lineage was isolated on LA plates. The selection of evolved isolates was based on increased colony size compared to the ancestral strain.

### PCR amplification and DNA sequencing

DNA amplification was performed using 2x PCR Mastermix (Thermo Scientific, Waltham, USA) according to the protocol of the manufacturer. Amplification products were purified using QIAquick PCR Purification Kit (Qiagen, Germany) and sequencing of purified PCR products was carried out by Macrogen (Amsterdam, The Netherlands). Sequences were analysed with the CLC Main Workbench 7.7.2 (CLCbio, Qiagen, Denmark).

Genomic DNA was prepared using the MasterPure DNA Purification Kit (Epicentre, Illumina Inc., Madison, Wisconsin) according to the manufacturer’s instructions and the samples were prepared for whole genome sequencing as according to Nextera® XT DNA Library Preparation Guide (Rev. D) (Illumina Inc., Madison, Wisconsin). Sequencing was performed using a MiSeq™ desktop sequencer, according to the manufacturer’s instructions (Illumina Inc., Madison, Wisconsin). Sequences were analysed with the CLC Genomic Workbench 11.0.0 (CLCbio, Qiagen, Denmark).
